# Management of Isolated Thoracic Lymphadenopathy of Unclear Etiology: A Survey of Physicians and Literature Review

**DOI:** 10.7759/cureus.41867

**Published:** 2023-07-14

**Authors:** Vikas Pathak, Nawaraj Adhikari, Courtney Conklin

**Affiliations:** 1 Pulmonary and Critical Care, Virginia Institute of Lung Diseases, Richmond, USA; 2 Internal Medicine, Bon Secours Memorial Regional Medical Center, Mechanicsville, USA; 3 Primary Care Sports Medicine, University of Alabama, Tuscaloosa, USA

**Keywords:** follow up, isolated lymph node, hilar lymph nodes, mediastinal lymph nodes, thoracic lymph nodes

## Abstract

Background

After identifying incidental mediastinal lymph nodes, decisions need to be made regarding the required follow-up imaging, the intervals at which this imaging should be performed, the types of imaging and procedures needed, and when to discontinue the follow-up. The purpose of this study is to determine the majority opinion on the management of these findings and provide recommendations for future management of incidental mediastinal lymphadenopathy.

Methodology

Sixty-two healthcare providers from a variety of specializations were surveyed on their preference for diagnostic workup and subsequent follow-up following the finding of incidental mediastinal lymphadenopathy on computed tomography (CT) of the chest.

Results

For thoracic lymphadenopathy of unclear etiology and patients who are not offered endobronchial ultrasound-guided transbronchial needle aspiration (EBUS-TBNA), most providers (47/62, 75.8%) initiate the CT scan follow-up at size 10 to 14 mm. Of those patients, 51.6% (32/62) of providers repeat the initial CT scan in three months and 41.9% (26/62) repeat the initial CT scan in six months. If the follow-up CT chest shows stable lymphadenopathy, 47.5% (29/62) repeat a CT chest every six months and 37% (23/62) repeat a CT chest every 12 months. The majority of providers (42/62, 67.7%) do not use positron emission tomography (PET)-CT for the initial evaluation of isolated thoracic lymphadenopathy and follow-up of lymphadenopathy with increasing size. For thoracic lymph nodes with a maximum diameter of 10 mm, only 4.8% (3/62) of providers continue CT screening after 24 months, while 24.6% (15/62) of providers continue CT screening after 24 months for sizes greater than 20 mm. Regarding the timing of EBUS-TBNA, 40.3% (25/62) of providers consider referring/performing this procedure at lymph nodes of size 11-15 mm, followed by 21% (13/62) of providers referring/performing the procedure at size 10 mm.

Conclusions

The majority of providers initiate CT scan follow-ups at 10 to 14 mm size for patients with isolated thoracic lymphadenopathy. The majority of providers do not use PET-CT for the initial evaluation of isolated thoracic lymphadenopathy. We found variable responses from providers regarding the timing of follow-up intervals and total duration. There is a need for consensus guidelines regarding the management of thoracic lymphadenopathy of unclear etiology.

## Introduction

A broad range of medical conditions, including pulmonary embolism, coronary artery disease, pleural disease, and other parenchymatous lung diseases, are evaluated with computed tomography (CT). With the increasing accessibility and usage of CT scans has come an increased prevalence of incidental findings, such as mediastinal lymphadenopathy. The prevalence of incidental mediastinal lymphadenopathy is reported to range from 0.15% to 3% [1-8]. The differential diagnosis for isolated mediastinal lymphadenopathy includes benign granulomatous disorders such as tuberculosis and sarcoidosis, malignancy, lymphoproliferative disorders, and reactive lymphadenopathy. Reactive lymphadenopathy can occur in response to chronic conditions such as emphysema and chronic bronchitis, interstitial lung disease, bronchiectasis, pulmonary arterial hypertension, heart failure, and connective tissue disease [9-13]. Mediastinal lymphadenopathy is present in 35% to 66% of patients with severe chronic cardiac failure [14-16]. With a wide range of differential diagnoses present for these incidental findings, providers are challenged with the decision of how to proceed after their discovery. Management of these findings varies across fields and providers.

## Materials and methods

A detailed questionnaire with 15 multiple questions was made regarding the practices of individual physicians on the follow-up of isolated mediastinal and or hilar lymphadenopathy. There were clear instructions on how to fill out the questionnaire.

Isolated thoracic lymphadenopathy was defined as mediastinal and or hilar lymphadenopathy of ≥10 mm (seen on CT chest on short axis) in size without any pulmonary parenchymal or pleural findings, absence of any known underlying malignancy and overall low risk of malignancy based on clinical evaluation. Patients aged <50 years, nonsmokers, with no personal history of cancer, and no first-degree relative with malignancy were considered low risk for malignancy.

The questionnaire was sent for review to the American Association of Bronchoscopy and Interventional Pulmonology (AABIP), which is a professional society, based in the United States for providers interested in the field of interventional pulmonology. Once reviewed and approved by the research committee of AABIP, the questionnaire was disseminated electronically to all the members of AABIP. The questionnaire was made using Google Forms, and all the results were automatically received by the research group. A total of 62 members replied to the questionnaire over three months.

Sixty-two medical professionals from a variety of backgrounds were surveyed on their management of incidental mediastinal lymphadenopathy. Providers surveyed included 20 who identified as specializing in pulmonary and critical care, seven who specialized in pulmonology, 34 who specialized in interventional pulmonology, and one who specialized in cardiothoracic surgery. Multiple countries of practice were present, including the United States, Canada, Brazil, Australia, and India. This survey consisted of 14 multiple-choice questions. Questions ranged from those regarding follow-up based on the size of lymph nodes, time intervals for repeating imaging, length of follow-up, imaging preferences, and use of endobronchial ultrasound-guided transbronchial needle aspiration (EBUS-TBNA).

## Results

Initial follow-up intervals

Following the initial finding of incidental mediastinal lymphadenopathy on CT imaging performed for other medical conditions, follow-up imaging is often recommended to track interval changes in findings. For those not offered EBUS-TBNA, the majority of providers (75%) initiate follow-up CT at 10 to 14 mm, 17% at 15 to 19 mm, and 6.5% at 20 mm and more (Table 1). The majority of surveyed providers (32/62, 51.6%) repeat the initial CT scan at three-month intervals, followed by providers who repeat imaging every six months (26/62, 41.9%). The longest interval offered, every 12 months, was selected by four of the 62 providers surveyed (Table 2).

**Table 1:** Diameter at which providers initiate CT scan follow-up for thoracic lymphadenopathy of unclear etiology. CT, computed tomography

Diameter of lymph nodes (mm)	Number of providers (Percentage), *n* (%)
10-14	47 (75.8)
15-19	11 (17.7)
>=20	4 (6.5)

**Table 2:** Interval for repeating the initial CT scan when incidental thoracic lymphadenopathy is noted. CT, computed tomography

Duration	Number of providers (Percentage), *n* (%)
Every 3 months	32 (51.6)
Every 6 months	26 (41.9)
Every 12 months	4 (6.5)

Follow-up of stable lymphadenopathy

Providers were surveyed on the length of follow-up required for stable lymphadenopathy of unknown etiology based on the maximum diameter of the thoracic lymph nodes. As summarized in Table 3, majority of the providers followed up with the second CT scan at six months (47.5%) or at 12 months (37.7%).

**Table 3:** Interval for repeating the second CT scan if the follow-up CT is stable in size. CT, computed tomography

Duration	Number of providers (Percentage), *n* (%)
Every three months	1 (1.6)
Every six months	29 (47.5)
Every nine months	4 (6.6)
Every 12 months	23 (37.7)
Every 18 months	2 (3.3)
Every 24 months	2 (3.3)

For lymph nodes with a maximum diameter of 10 mm, the majority of providers (26/62, 41.9%) discontinued CT screening after 24 months with no change in diameter followed by 33.9% (21/62) of providers who discontinued screening after 12 months. For lymph nodes with a maximum diameter of 11 to 15 mm, the majority of providers (32/62, 51.6%) discontinued CT screening after 24 months with no change in diameter followed by 30.6% (19/62) of providers who discontinued screening after 12 months.

For lymph nodes with a maximum diameter of 16 to 20 mm, the majority of providers (39/62, 62.9%) discontinued CT screening after 24 months with no change in diameter followed by 19.4% (12/62) of providers who discontinued screening after 12 months.

For lymph nodes with a maximum diameter of greater than 20 mm, the majority of providers (37/62, 59.7%) discontinued CT screening after 24 months with no change in diameter followed by 24.6% (15/62) of providers who continued screening longer than 24 months (Table 4).

**Table 4:** Time length to discontinue CT screening based on different sizes of thoracic lymphadenopathy when the diameter remains unchanged or with minimal change. CT, computed tomography

Time length to discontinue CT	Number of providers (Percentage), *n* (%)			
	10 mm	11-15 mm	16-20 mm	>20 mm
After six months	6 (9.7%)	4 (6.5%)	2 (3.2%)	1 (1.6%)
After 12 months	21 (33.9%)	19 (30.6%)	12 (19.4%)	8 (13.1%)
After 18 months	6 (9.7%)	4 (6.5%)	2 (3.2%)	0 (0.0%)
After 24 months	26 (41.9%)	32 (51.6%)	39 (62.9%)	37 (60.7%)
Longer than 24 months	3 (4.8%)	3 (4.8%)	7 (11.3%)	15 (24.6%)

Use of PET-CT

PET-CT for the initial evaluation of isolated thoracic lymphadenopathy was not commonly used by providers. Of the 62 providers, 42 did not use PET-CT for evaluation before EBUS-TBNA.

In the setting of thoracic lymph nodes, which were increasing in size, 67.7% (42/62) providers did not routinely order PET-CT imaging before performing EBUS-TBNA or mediastinoscopy. The diameter of the lymph node at which providers would consider doing PET-CT before referral for EBUS-TBNA or mediastinoscopy is summarized in Table 5.

**Table 5:** Diameter at which providers consider doing PET-CT before referral for EBUS-TBNA or mediastinoscopy for lymph nodes of increasing size. PET-CT, positron emission tomography-computed tomography; EBUS-TBNA, endobronchial ultrasound-guided transbronchial needle aspiration

Diameter of lymph nodes (mm)	Number of providers (Percentage), *n* (%)
<10	2 (3.3)
10	16 (26.7)
11-15	29 (48.3)
16-20	8 (13.3)
>20	5 (8.3)

Use of EBUS-TBNA

When surveyed on when to proceed with EBUS-TBNA, 25/62 providers stated that once lymph nodes reached a diameter of 11 to 15 mm, they would refer/perform EBUS-TBNA or mediastinoscopy. This was followed by 20.9% (13/62) who answered 10 mm and 19.4% (12/62) who responded with 16 to 20 mm (Table 6).

**Table 6:** Diameter at which providers consider referring/performing an endobronchial ultrasound or mediastinoscopy to determine the etiology.

Diameter of lymph nodes (mm)	Number of providers (Percentage), *n* (%)
<10	3 (4.8)
10	13 (21)
11-15	25 (40.3)
16-20	12 (19.4)
>20	9 (14.5)

Of the 62 providers surveyed, 57 would perform EBUS-TBNA to determine the etiology of a thoracic lymph node. Of the five who would not perform EBUS-TBNA, one specialized in pulmonary and critical care and referred about six to 10 patients per month for this procedure, two specialized in pulmonology and referred about zero to five patients per month, and two specialized in pulmonary and critical care and referred about zero to five patients per month. A majority of the providers surveyed (44/62, 70.9%) said they referred zero to five patients per month for EBUS-TBNA to determine the etiology of the enlarged lymph node followed by 20.9% (13/62) of providers who referred six to 10 times per month for EBUS-TBNA. Two providers surveyed stated they referred over 20 patients per month for this procedure.

## Discussion

In our study, for the patients not offered EBUS-TBNA, 75% of surveyed providers initiate follow-up CT scan at the smallest size (10-14 mm). Thoracic lymphadenopathy is considered abnormal when greater than 10 mm in the short axis. The American College of Radiology (ACR) Incidental Findings Committee recommends no further workup for mediastinal lymph nodes less than 15 mm with benign features in asymptomatic patients [17]. In a study done in 100 isolated mediastinal lymphadenopathies undergoing EBUS-TBNA, all patients with lymph nodes size 15 mm or less (*n* = 19) were reactive [18]. The majority of providers (51%) repeated the initial CT at three months and 41% repeated it at six months. The majority of providers discontinued CT screening after 24 months if the size remained constant during the follow-up. In a study that included 82 patients undergoing EBUS-TBNA for mediastinal lymphadenopathy follow-up CT was available for 36/62 patients with no classifying diagnosis [19]. The scans were done at a median duration of 118 days (10-692 days) and only 2/36 patients had lymphadenopathy progressed in size and number.

In our study, providers followed up longer for lymphadenopathy with bigger sizes although they remained constant in size. There is not enough data regarding the risk of malignancy with increasing size in isolated mediastinal lymphadenopathy. So, providers likely practice based on other similar disease conditions. A meta-analysis that included patients with non-small-cell lung cancer showed an increased risk of malignancy with increasing size of the lymph node [20]. In a retrospective study on patients who underwent low-dose CT for lung cancer screening, over seven years, patients with mediastinal lymphadenopathy had a higher incidence of lung cancer compared to those without mediastinal lymphadenopathy (17% versus 3.9%, *P* < 0.001) [21]. This study recommended follow-up imaging within one year for patients with mediastinal lymphadenopathy in low-dose screening CT.

A significant portion (32.3%) of surveyed providers used PET-CT before performing EBUS-TBNA or mediastinoscopy. However, PET-CT has limited value in mediastinal lymphadenopathy with no concern for malignancy. In a study including 100 patients with mediastinal lymphadenopathy, of the 29 patients with PET-CT, 25 had increased F-fluorodeoxyglucose (FDG) uptake; however, none of them had malignancy in biopsy results [19]. Prior studies have shown that granulomatous conditions like tuberculosis and sarcoidosis may also have increased FDG uptake [22-23]. Due to this, there is a concern for a high false-positive rate with PET-CT.

Around 40% of surveyed providers consider referring/performing endobronchial ultrasound/mediastinoscopy at the size of 11-15 mm. EBUS-TBNA is the initial investigation of choice for patients with isolated thoracic lymphadenopathy requiring pathologic diagnosis. In a prospective study including 77 patients with isolated lymphadenopathy, EBUS-TBNA had a high sensitivity of 92% and a low negative predictive value (NPV) of 40% [24]. This study showed that EBUS-TBNA is a cost-effective procedure and can prevent 87% of mediastinoscopy. The prevalence of reactive lymphadenopathy was very low (5%) in this study, which does not represent the general population and could have given the low NPV. In another study, including 100 patients with isolated mediastinal lymphadenopathy, EBUS-TBNA had a sensitivity of 82.7% and an NPV of 84.2%.

The workup for isolated thoracic lymphadenopathy starts with a careful history and physical examination. Demographic factors such as age, occupation, smoking history, area of residence, and other comorbidities help formulate the differential diagnosis. Once the mediastinal lymph node is confirmed in a CT scan, it is important to find the accurate mediastinal station involved and also note its size and characteristics as homogenous, symmetric/asymmetric, presence of calcification, etc. [25]. A CT scan can also help find the underlying lung disease as the etiology of lymphadenopathy. Based on the location of lymph nodes, the decision can be made if bronchoscopic modalities can be used for pathologic diagnosis. EBUS-TBNA is currently considered a first-line diagnostic modality and has replaced invasive mediastinoscopy [25]. ACR Incidental Findings Committee published its recommendation on the management of incidental thoracic lymphadenopathy in 2018 [17]. For asymptomatic mediastinal lymphadenopathy less than 15 mm, ACR recommends no further workup. For lymph nodes greater than or equal to 15 mm with no explainable disease, it recommends clinical consultation and/or PET-CT and/or three to six months follow-up chest CT. For those with increased size in follow-up, ACR recommends PET-CT or biopsy, and for those with stable or decreased size, no further workup is recommended. However, PET-CT can be positive in many benign conditions and the use of PET-CT for isolated mediastinal lymphadenopathy is debatable unless there is a concern for some underlying primary cancer. We believe it is reasonable to follow up on isolated mediastinal lymphadenopathy with a CT scan and perform EBUS-TBNA for the lymph nodes with increasing size.

Our study had a few limitations. We used convenience sampling for our survey, which is subject to selection bias. Our sample size was small as only 62 providers participated in our study. The survey questions aimed at assessing the practice of the providers, and there could be some recall bias.

## Conclusions

The majority of providers initiate follow-up CT scan at 10 to 14 mm, although the prior study has suggested no further workup is needed for lymph nodes less than or equal to 15 mm. Most providers tend to continue follow-up CT scans for a longer duration for thoracic lymph nodes of larger size although the size remains unchanged or with minimal change. The majority of providers do not consider PET-CT for the initial evaluation of isolated thoracic lymphadenopathy and also for thoracic lymph nodes with increasing size before performing EBUS-TBNA or mediastinoscopy. Providers have variable responses regarding the timing interval of initial CT and follow-up CT. A few studies have provided limited recommendations on the management of isolated mediastinal lymphadenopathy. There is a need for a comprehensive and consensus guideline for the management of incidental mediastinal lymphadenopathy and its follow-up.
